# Correlation of serum HMGB1 and HMGB2 levels with clinical symptoms in allergic rhinitis children

**DOI:** 10.1097/MD.0000000000034921

**Published:** 2023-09-15

**Authors:** Xinxin Xing, Hai Wang

**Affiliations:** a Department of Pediatrics II, First Affiliated Hospital of Heilongjiang University of Chinese Medicine, Xiangfang District, Harbin, China.

**Keywords:** allergic rhinitis, HMGB1, HMGB2, inflammatory factors

## Abstract

This research aimed to explore the serum high-mobility group box 1 (HMGB1) and high-mobility group box 2 (HMGB2) levels in allergic rhinitis (AR) children and its correlation with clinical results. This present prospective observational study enrolled 179 AR children and 100 healthy children who came to our hospital during October 2020 to August 2022. The serum HMGB1, HMGB2, interleukin (IL)-6, IL-1β, interferon-γ, and C-reactive protein (CRP) levels were measured by enzyme-linked immunosorbent assay. Demographic and clinical statistics including age, body mass index (BMI), sex, diastolic blood pressure, SBP, family history of allergy, Visual Analogue Score (VAS) and Rhinoconjunctivitis Quality of Life Questionnaire were collected. All data used SPSS 18.0 to statistical analyses. The proportion of family history of allergy was obviously higher in the AR group than that in the healthy group. The serum levels of HMGB1, HMGB2 and cytokines were remarkably enhanced in the AR patients. Spearman analysis supported that positive correlation existed among the HMGB1, HMGB2, CRP, IL-6 and IL-1β levels. Serum IL-6, CRP, HMGB2, IL-1β, VAS score and Rhinoconjunctivitis Quality of Life Questionnaire score levels were significantly higher and serum interferon-γ levels were significantly lower in the HMGB1 high expression group. Similar results were found in in the HMGB2 high group compared to the HMGB2 low group. In addition, HMGB1 and HMGB2 could be potential diagnostic biomarkers of AR patients. Finally, we found that HMGB1, HMGB2, IL-6, IL-1β, and family history of allergy were the risk factors for AR. This study showed that the serum HMGB1 and HMGB2 levels was remarkably enhanced in AR patients and closely associated with cytokines. This study may provide new targets and a comprehensive approach for the treatment of AR patients.

## 1. Introduction

Allergic rhinitis (AR) is one of the common immunological disorders with clinical symptoms such as frequent episodes of sneezing, clear nasal discharge, and significant nasal congestion.^[1]^ With the growing worldwide prevalence of AR, about 10% to 20% of people in the world are affected by AR.^[2]^ According to recent estimates from the Agency for Healthcare Research and Quality, the prevalence of AR in adults ranges from 10% to 30%, but its prevalence in children is already approaching 40%.^[3]^ The pathological mechanism of AR is complex,^[4]^ and risk factors for AR have been reported to be related to genetic,^[5]^ environmental,^[6]^ psychological,^[7]^ and regional differences, etc.^[8,9]^ AR predisposes or exacerbates patients to fatigue, headaches, cognitive impairment, and sleep disturbances and is a risk factor for asthma.^[10]^ However, the diagnosis of AR still relies on observation of patients’ symptoms,^[11]^ allergen skin testing, and lack of specific serum markers.

High-mobility group box 1 (HMGB1) is a nonhistone DNA-binding protein widely found in the nucleus of eukaryotic cells.^[12]^ HMGB1 can be released into the extracellular space from neurons or glial cells to induce immune cell activation and cause inflammatory response.^[13]^ While the amino acid sequence of HMGB2 is highly similar to that of HMGBl, more than 80% of its amino acid sequences are identical.^[14]^ HMGB2 is present in large numbers in immune cells, which can damage the intima and lead to hyperplasia of the intima.^[15]^ Previous reports had demonstrated that overexpression of HMGB1 and HMGB2 is associated with the severity and poor prognosis of a number of inflammatory diseases and cancer.^[15,16]^ HMGB1 has been found to play an important mechanism in the pathogenesis of AR in animal studies.^[17,18]^ However, so far, no clinical studies focus on the role of HMGB1 and HMGB2 in AR development.

In the present prospective observational research, we aimed to explore the expression of HMGB1 and HMGB2 in AR patients and its correlation with clinical results. This study might reveal the clinical significance of HMGB1 and HMGB2 in AR patients, as well as provide novel research targets for ischemic stroke treatment.

## 2. Methods

### 2.1. Subjects

This present prospective observational study enrolled 179 allergic rhinitis (AR) children who came to our hospital during October 2020 to August 2022. All patients were diagnosed AR according to BSACI guidelines for the diagnosis and management of allergic and nonallergic rhinitis,^[11]^ with specific inclusion criteria: (1) Sneezing, clear watery nose, nasal itching, and nasal congestion present for 2 or more times. Symptoms persist or accumulate for more than 1 hour per day with other concomitant disease symptoms such as respiratory symptoms (including cough, wheezing, etc) and ocular symptoms (including itching, tearing, redness, and burning sensation of the eyes); (2) pale and edematous nasal mucosa with watery nasal discharge is common; (3) allergen testing for at least 1 allergen with positive serum-specific IgE; (4) allergen skin test showing dust mite punctate wind cluster ≥ 3 mm in diameter; (5) age < 14. The exclusion criteria included: (1) persons with other allergic diseases or autoimmune diseases; (2) patients with a history of chronic diseases such as diabetes and hypertension; (3) patients who had used hormones, immunosuppressive or enhancing, anti-inflammatory drugs etc within 7 days prior to enrollment; (4) patients with seriously infection, severe liver, renal, malignancy, cardiovascular dysfunctions; (5) patients with a history of nasal masses, nasal polyps, sinusitis, etc. In addition, we collected serum samples and clinical data from 100 healthy children. All participants signed a written informed consent and were followed up for 3 months. The study was approved by the Ethics Committee of the author’s hospital.

### 2.2. Blood sampling measurement

The serum HMGB1, HMGB2, interleukin (IL)-6, IL-1β, interferon-γ (IFN-γ), and C-reactive protein (CRP) levels were measured by enzyme-linked immunosorbent assay (ELISA). Blood samples of fasting cubital venous (5 mL) were collected within 24 hours after admission for all cases. Samples were centrifuged at 2000 g for 15 minutes, following with ELISA tested using commercially available kits (HMGB1 MBS3803280 MyBioSource, HMGB2 MBS763599 MyBioSource, IL-6 MBS175877 MyBioSource, CRP MBS177184 MyBioSource, IL-1β MBS175901 MyBioSource, IFN-γ EK0373 BOSTER).

### 2.3. Data collection and scale scoring

Demographic and clinical statistics including age, BMI, sex, diastolic blood pressure, systolic blood pressure, family history of allergy, etc were collected. Visual Analogue Score (VAS) and Rhinoconjunctivitis Quality of Life Questionnaire were used to assess the patient’s symptom profile.

### 2.4. Statistical analysis

The normal distribution of data was confirmed by Kolmogorov–Smirnov analysis. Normal distribution data were expressed by mean ± SD while non-normal distribution data median (range). Mann–Whitney test or Student *t* test was used for comparison between 2 groups. Chi square test was used for rates. Spearman rank correlation was used for correlation analysis. ROC curves were used to analyze the diagnostic value of HMGB1 for AR. Logistic regression was performed for risk factors of AR. *P* < .05 regarded significant difference. All data used SPSS 18.0 to statistical analyses.

## 3. Results

### 3.1. Clinical characteristics of all participants

This study enrolled 179 AR patients and 100 healthy children. The basic information was shown in Table 1. Compared the demographic and clinical data of 2 groups, we found that the proportion of family history of allergy was obviously higher in the AR group than that in the healthy group (*P* < .05). No other significantly difference was found in age, sex, BMI, SBP, and diastolic blood pressure.

**Table 1:** Demographic and clinical data of all subjects.

Variable	AR children, n = 179	Healthy children, n = 100	*P*
Age, y	8 (3–14)	9 (3–14)	.952
Sex, female (%)	81 (45.25)	41 (41)	.668
BMI	22.33 (17.89–26.36)	22.27 (17.89–26.30)	.692
SBP (mm Hg)	99.48 ± 10.34	99.75 ± 9.68	.819
DBP (mm Hg)	68.94 ± 6.33	68.59 ± 5.77	.648
Family history of allergy, n (%)	68 (37.99)	14 (14)	<.001
VAS	3.87 ± 1.42		
RQLQ	28.08 ± 7.16		

AR = allergic rhinitis, BMI = body mass index, DBP = diastolic blood pressure, RQLQ = Rhinoconjunctivitis Quality of Life Questionnaire, SBP = systolic blood pressure, VAS = visual analogue score.

### 3.2. Serum levels of HMGB1, HMGB2, and inflammatory factors in AR patients

**Figure 1.**
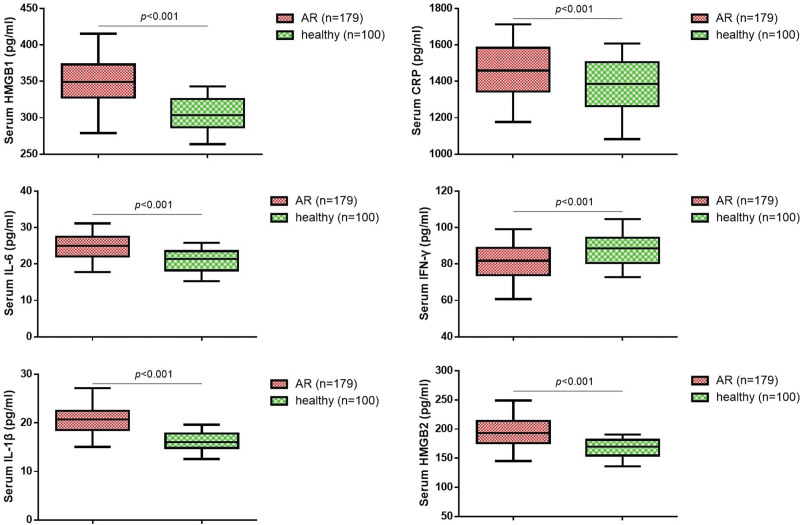
Comparisons of HMGB1, HMGB2 and inflammatory factors in all subjects. HMGB1 = high-mobility group box 1, HMGB2 = high-mobility group box 2.

To further investigate the relationship between HMGB1, HMGB2, and inflammatory in AR patients, we measured the serum levels of HMGB1, HMGB2, IL-6, IFN-γ, IL-1β, and CRP by ELISA. As shown in Figure 1, the serum levels of HMGB1, HMGB2, IL-6, IL-1β, and CRP were remarkably enhanced in the AR patients compared with healthy persons. However, the serum levels of IFN-γ in AR group were significantly declined compared with healthy group. Spearman analysis supported that positive correlation existed among the HMGB1, HMGB2, IL-6, and IL-1β levels (Table 2). In addition, we also found IFN-γ was negatively correlated with HMGB1, HMGB2, CRP, IL-6, and IL-1β.

**Table 2:** Correlation analysis among HMGB1, HMGB2, and cytokines.

	HMGB1	CRP	IL-6	IFN-γ	IL-1β	HMGB2
HMGB1						
Spearman correlation	1	0.115	0.424	–0.293	0.460	0.431
*P*		.055	<.001	<.001	<.001	<.001
CRP						
Spearman correlation	0.115	1	0.068	–0.207	0.109	0.103
*P*	.055		.257	.001	.070	.085
IL-6						
Spearman correlation	0.424	0.068	1	–0.294	0.465	0.413
*p*	<.001	.257		<.001	<.001	<.001
IFN-γ						
Spearman correlation	–0.293	–0.207	–0.294	1	-0.357	-0.290
*P*	<.001	.001	<.001		<.001	<.001
IL-1β						
Spearman correlation	0.460	0.109	0.465	–0.357	1	0.456
*P*	<.001	.070	<.001	<.001		<.001
HMGB2						
Spearman correlation	0.431	0.103	0.413	–0.290	0.456	1
*P*	<.001	.085	<.001	<.001	<.001	

CRP = C-reactive protein, HMGB1 = high-mobility group box 1, HMGB2 = high-mobility group box 2, IFN-γ = interferon-γ, IL = interleukin.

### 3.3. Correlation between serum HMGB1, HMGB2 levels and AR patients’ clinical outcome

Further, all patients were divided into HMGB1 high expression group and low expression group according to its mean value (350.65 pg/mL) and the clinical characteristics were compared. As summarized in Table 3, the serum levels of CRP, IL-6, HMGB2, and IL-1β levels in HMGB1 high expression group were remarkably enhanced compared with the patients with low HMGB1 levels (*P* < .05). In addition, the serum IFN-γ levels in HMGB1 high expression group were obviously decreased (*P* < .05). Besides, compared with HMGB1 high expression group, the VAS scores and Rhinoconjunctivitis Quality of Life Questionnaire scores were significantly higher than that patients in the HMGB1 low expression group(*P* < .05), suggesting that HMGB1 was associated with the clinical outcomes and severity in AR patients. We also divided all patients into HMGB2 high expression group and low expression group according to the median HMGB2 value (195.92 pg/mL) to compare the clinical data. As shown in Table 4, we obtained similar results. However, no significantly difference was found in IL-6 and CRP levels between 2 groups.

**Table 3:** Correlation between serum HMGB1 levels and the clinical outcome of AR patients.

Variables	HMGB1 low, n = 94	HMGB1 high, n = 85	*P*
Age, y	8 (3–14)	9 (3–14)	.997
Sex, female (%)	43 (45.74)	38 (44.71)	.999
Family history of allergy, n (%)	40 (42.55)	28 (32.94)	.190
BMI	22.65 (17.98–26.23)	22.01 (17.89–26.36)	.424
SBP (mm Hg)	99.66 ± 10.10	99.25 ± 10.58	.792
DBP (mm Hg)	68.71 ± 6.50	69.20 ± 6.17	.607
VAS	3.4 (1.8–7.0)	4.0 (1.9–7.0)	.020
RQLQ	29 (18–43)	25 (16–43)	.001
CRP (pg/mL)	1430.25 ± 132.61	1477.14 ± 129.11	.018
IL-6 (pg/mL)	24.34 ± 3.26	25.37 ± 3.29	.038
IFN-γ (pg/mL)	84.67 (60.79–99.09)	78.89 (61.72–98.61)	.014
IL-1β (pg/mL)	20.26 ± 2.95	21.17 ± 2.79	.036
HMGB2 (pg/mL)	191.49 ± 24.82	200.81 ± 26.25	.016

BMI = body mass index, CRP = C-reactive protein, DBP = diastolic blood pressure, HMGB1 = high-mobility group box 1, IFN-γ = interferon-γ, IL = interleukin, RQLQ = Rhinoconjunctivitis Quality of Life Questionnaire, SBP = systolic blood pressure, VAS = visual analogue score.

**Table 4:** Correlation between serum HMGB2 levels and the clinical outcome of AR patients.

Variables	HMGB2 low, n = 93	HMGB2 high, n = 86	*P*
Age, y	8 (3–14)	9 (3–14)	.388
Sex, female (%)	46 (49.46)	35 (40.70)	.320
Family history of allergy, n (%)	34 (36.56)	34 (39.53)	.771
BMI	22.17 (17.95–26.26)	22.48 (17.89–26.36)	.312
SBP (mm Hg)	99.91 ± 10.24	99.00 ± 10.41	.553
DBP (mm Hg)	69.56 ± 6.05	68.26 ± 6.59	.170
VAS	3.2 (1.8–6.7)	4.1 (2.2–7.0)	<.001
RQLQ	30 (16–43)	24 (16–42)	<.001
CRP (pg/mL)	1457.89 ± 137.64	1446.70 ± 127.66	.574
IL-6 (pg/mL)	24.74 (17.76–31.01)	25.56 (17.95–31.15)	.084
IFN-γ (pg/mL)	84.72 (61.10–99.09)	79.17 (60.79–98.61)	.022
IL-1β (pg/mL)	19.98 ± 2.82	21.47 ± 2.81	.001
HMGB1 (pg/mL)	344.36 ± 35.27	357.46 ± 29.54	.008

BMI = body mass index, CRP = C-reactive protein, DBP = diastolic blood pressure, HMGB1 = high-mobility group box 1, IFN-γ = interferon-γ, IL = interleukin, RQLQ = Rhinoconjunctivitis Quality of Life Questionnaire, SBP = systolic blood pressure, VAS = visual analogue score.

### 3.4. Diagnostic value of HMGB1 and HMGB2 of AR patients

**Figure 2.**
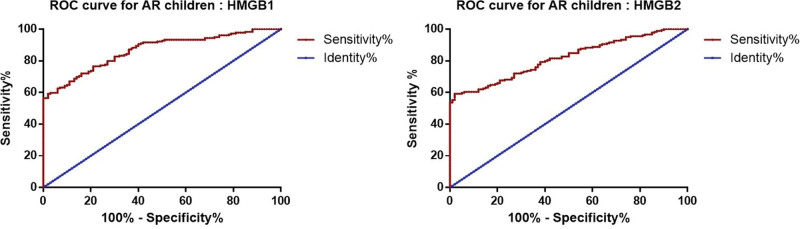
ROC curves for HMGB1 and HMGB2 in diagnostic of AR. AR = Allergic rhinitis, HMGB1 = high-mobility group box 1, HMGB2 = high-mobility group box 2.

We draw ROC curves to assess the diagnostic value of HMGB1 and HMGB2 for AR patients. The result showed that HMGB1 and HMGB2 could be potential diagnostic biomarkers of AR patients and HMGB1 had better diagnostic value (Fig. 2), the AUC of HMGB1 was 0.865, cutoff value 326.68 pg/mL, sensitivity 76.5%, specificity 79.0% and the AUC of HMGB2 was 0.817, cutoff value 179.65 pg/mL, sensitivity 72.1%, specificity 73.0%.

### 3.5. Risk factors of AR patients by logistic regression analysis

Finally, we used entry method for logistic regression to analyze the risk factors of AR. For logistic regression, we used the entry method. It was found that HMGB1, HMGB2, IL-6, IL-1β, and family history of allergy were the risk factors for AR (Table 5).

**Table 5:** Logistic regression for risk factors of AR.

Variables	Wald	Odds ratio	95% CI	*P*
Age	0.033	1.014	0.869–1.185	.856
Sex	0.002	1.026	0.348–3.029	.962
BMI	0.078	1.034	0.817–1.310	.780
Family history of allergy	10.312	0.113	0.030–0.428	.001
SBP	0.040	0.994	0.941–1.051	.842
DBP	0.020	1.007	0.916–1.107	.888
CRP	2.779	0.997	0.993–1.001	.095
IL-6	5.584	0.810	0.681–0.965	.018
IFN-γ	0.336	1.019	0.955–1.087	.562
IL-1β	31.825	0.445	0.336–0.589	<.001
HMGB1	20.554	0.943	0.919–0.967	<.001
HMGB2	10.099	0.947	0.919–0.979	.001

AR = Allergic rhinitis, BMI = body mass index, CRP = C-reactive protein, DBP = diastolic blood pressure, HMGB1 = high-mobility group box 1, HMGB2 = high-mobility group box 2, IFN-γ = interferon-γ, IL = interleukin, SBP = systolic blood pressure, VAS = visual analogue score.

## 4. Discussion

The symptoms of AR may often disrupt patients’ sleep and affect their work and school, causing significant negative impact on quality of life and leading to significant social costs. In China, the incidence of AR has remained on the rise in recent years, with a significant impact on the public, especially children.^[19]^ Thus, it is urgent to develop new biomarkers and comprehensive approaches to prompt diagnosis and early treatment of AR. In this research, we found that the serum levels of HMGB1 and HMGB2 were increased in AR patients and were associated with patients’ clinical outcomes and severity.

Several studies have similarly focused on serum biomarkers in AR patients. A clinical study by Han et al confirmed that serum IL-1β could be a biomarker of AR and correlated with disease severity.^[20]^ Han’s findings are similar to ours, in which IL-1β was also found to be elevated in the serum of AR patients and correlated with other cytokines. Some earlier studies likewise found that the inflammatory factors IL-17 as well as TNF-α were associated with the development of AR.^[20,21]^ We also investigated the correlation between IFN-γ and inflammatory factors and found that IFN-γ was negatively correlated with IL-6 and IL-1β, verifying the results of Han et al, and some cytokines (IL-17, IL-22, and IL-35) were also found to be closely associated with disease development in AR in their study.^[22]^ In the last 2 years of research, the clinical significance of some new biomarkers in AR has also been noticed. Wen et al supported that serum nitric oxide synthase levels were elevated in AR patients and could be used to predict the treatment of AR.^[23]^ Ramin et al suggested that serum levels of resolvin E1 and leukotriene B4 were significantly higher in AR patients than healthy persons.^[24]^ However, up to now, there are no specific biomarker for the diagnosis of AR.

HMGB protein family has been confirmed to be involved in cellular proliferation, apoptosis, oxidative stress and inflammatory responses in a variety of diseases.^[25–27]^ In autoimmune diseases, Tsakalidou et al demonstrated that serum HMGB1 levels correlated with disease activity and severity in systemic lupus erythematosus patients.^[28]^ A review by Taniguchi et al found that multiple functions of the HMGB protein family (HMGB1, 2, 3, and 4) play a complex role as intrinsic and endogenous regulators in rheumatoid arthritis. Several previous studies have also focused on the relationship between HMGB1 and AR. Yue et al found that inhibition of HMGB1 improved allergic rhinitis as well as inflammatory factor expression in mice model.^[18]^ Yuan et al suggested that SIRT1 administration attenuated HMGB1 protein expression and regulated the production of pro-inflammatory mediators to reduce allergic symptoms in AR mice.^[29]^ A clinical study by Zhu et al found high expression of HMGB1 levels in nasal lavage fluid samples from AR patients.^[30]^ However, there are no more clinical studies on the role of HMGB1 and HMGB2 in patients with AR. In our study, we found that serum HMGB1 and HMGB2 levels were declined in AR patients and were associated with other inflammatory factors.

This present research also has some limitations. First, we only included a small size of study population. Secondly, we only checked a small number of inflammatory factors. Thirdly, the molecular mechanism of HMGB1 and HMGB2 affecting AR development is unclear.

## 5. Conclusion

This study showed that the serum HMGB1 and HMGB2 levels was remarkably enhanced in AR patients. In addition, HMGB1 and HMGB2 were closely associated with cytokines and clinical outcomes in AR patients. This study may provide new targets and a comprehensive approach for the treatment of AR patients.

## Author contributions

**Data curation:** Hai wang.

**Formal analysis:** Xinxin Xing.

**Writing – original draft:** Xinxin Xing, Hai Wang.

**Writing – review & editing:** Hai Wang.
